# Insoluble and Thermostable Polyhydroxyesters From a Renewable Natural Occurring Polyhydroxylated Fatty Acid

**DOI:** 10.3389/fchem.2019.00643

**Published:** 2019-09-24

**Authors:** José Jesús Benítez, Susana Guzman-Puyol, Miguel Antonio Cruz-Carrillo, Luca Ceseracciu, Ana González Moreno, Antonio Heredia, José Alejandro Heredia-Guerrero

**Affiliations:** ^1^Instituto de Ciencia de Materiales de Sevilla, Centro Mixto CSIC-Universidad de Sevilla, Seville, Spain; ^2^Instituto de Hortofruticultura Subtropical y Mediterránea La Mayora, Departamento de Biología Molecular y Bioquímica, Universidad de Málaga, Málaga, Spain; ^3^Instituto de Hortofruticultura Subtropical y Mediterránea La Mayora, Universidad de Málaga-CSIC, Málaga, Spain; ^4^Facultad de Ingeniería Culiacán, Universidad Autónoma de Sinaloa, Culiacán, Mexico; ^5^Materials Characterization Facility, Istituto Italiano di Tecnologia, Genova, Italy

**Keywords:** renewable fatty acids, aleuritic acid, long-chain polyesters, food packaging, melt-polycondensation

## Abstract

To explore the potential of long chain polyhydroxyalkanoates as non-toxic food packaging materials, the characterization of polyesters prepared from a natural occurring polyhydroxylated C16 carboxylic acid (9,10,16-trihydroxyhexadecanoic or aleuritic acid) has been addressed. Such monomer has been selected to elucidate the reactivity of primary and secondary hydroxyl groups and their contribution to the structure and properties of the polyester. Resulting polyaleuritate films have been produced using an open mold in one-step, solvent-free self-polycondensation in melt state and directly in air to evaluate the effect of oxygen in their final physical and chemical properties. These polymers are amorphous, insoluble, and thermostable, being therefore suitable for solvent, and heat resistant barrier materials. Structurally, most of primary hydroxyls are involved in ester bonds, but there is some branching arising from the partial participation of secondary O-H groups. The oxidative cleavage of the vicinal diol moiety and a subsequent secondary esterification had a noticeable effect on the amorphization and stiffening of the polyester by branching and densification of the ester bond network. A derivation of such structural modification was the surface compaction and the reduction of permeability to water molecules. The addition of Ti(OiPr)_4_ as a catalyst had a moderate effect, likely because of a poor diffusion within the melt, but noticeably accelerated both the secondary esterification and the oxidative processes. Primary esterification was a high conversion bulk reaction while oxidation and secondary esterification was restricted to nearby regions of the air exposed side of cast films. The reason was a progressive hindering of oxygen diffusion as the reaction progresses and a self-regulation of the altered layer growth. Despite such a reduced extent, the oxidized layer noticeably increased the UV-vis light blockage capacity. In general, characterized physical properties suggest a high potential of these polyaleuritate polyesters as food preserving materials.

## Introduction

The environmental and health threat caused by massive production, use, and disposal of fuel-based synthetic polymers is a major issue of concern in our society (Lithner et al., 2011). More recently, such worry has been focused on the accumulation and impact of single use packaging plastics on freshwater and marine ecosystems and the consequences for human health (Law, 2017; Blettler et al., 2018; Hamid et al., 2018). The global strategy to palliate the problem is based on the closed “reduce-recycle-reuse” loop of circular economy. While these concepts rely on a social consciousness-raising, a fourth pillar “replace” has driven the quest for sustainable and eco-friendly bio-based materials substituting fossil-based polymers (Shen et al., 2010; Chen and Patel, 2012; Vilela et al., 2014; Gandini and Lacerda, 2015). Thus, solutions based on an abundant and renewable plant feedstock such as cellulose and lignocellulosics have been proposed over these years (Klemm et al., 2005; Brodin et al., 2017). Among other bioplastics, short chain polyesters such as poly(lactic acid) (PLA), polycaprolactone (PCL), and polyhydroxyalkanoates (PHVs, PHBs, etc.) have gathered particular interest due to their biocompatibility and biodegradability. However, much less attention has been paid to long chain homologs, though the lower ester bond/mass ratio would facilitate their degradability rate and longer alkyl chain would improve hydrophobicity and thermal stability. The main reason behind this failure is the difficulty in designing a cost-effective and environmentally friendly route to the production of fatty hydroxyacid monomers. Most of the synthetic methods to prepare fatty ω-hydroxyacids use vegetable oils as feedstock (Petrović et al., 2010; Jose et al., 2014), though biosynthetic ways have also been reported (Lu et al., 2010; Liu et al., 2011). However, fatty ω-hydroxy and polyhydroxyacids (C16 and C18) are already present in Nature as monomers of barrier biopolymers such as cutin and suberin in higher plant (Domínguez et al., 2011; Graça, 2015). With an appropriate processing, this natural abundant and renewable resource can be exploited as a profitable feedstock for the synthesis of fatty polyhydroxyalkanoates for specific applications (Gandini et al., 2006; Heredia-Guerrero et al., 2017b). In particular, cutin monomers from tomato fruits have been used in the preparation of films and coatings for cosmetics, biomedicine, and food packaging (Heredia-Guerrero et al., 2017b).

Monomers conforming the biopolyester cutin are mostly C16 and C18 hydroxyacids, commonly midchain hydroxylated ω-hydroxyacids (Baker and Holloway, 1970; Walton and Kolattukudy, 1972), but there are other chemical groups such as unsaturations, epoxy, and secondary vicinal diols. This later functionality is also present in aleuritic (9,10,16-trihydroxyhexadecanoic) acid, the main component of shellac, a natural resin with applications in food, pharmaceutics and coatings and a potential production of 50,000 tons/year (Heredia-Guerrero et al., 2017a). Aleuritic acid is an excellent model molecule to study the role of primary and secondary hydroxyl groups and the contribution of the vicinal diol moiety to the structure and properties of the polyester formed by self-esterification. Previous work has been focused on the viability of the reaction in these particular conditions (Benítez et al., 2015a,b) and in the regulation of the OH/COOH imbalance by the addition of palmitic acid (Benítez et al., 2015c, 2016). In this article, the study is expanded to a much broader range of temperature and reaction time to set the optimal polymerization conditions, and the extent of the side reactions observed. Though intended to be a catalyst free route, here a common esterification catalyst such as Ti(OiPr)_4_ has also been used to evaluate its convenience.

Though natural occurring fatty hydroxyacids from plant cutin or shellac are still far from being a cost-effective feedstock for bulk packaging materials, the perspective may change when designing few microns-thick coatings in which the amount of material per container is reduced by 2–3 orders of magnitude. In this scenario, aspects such as non-toxicity would make up for cost, as for instance, in the replacement of BPA internal varnishes in food cans.

Thus, to design a non-toxic and easily scalable synthesis route to a one-step preparation of thin polyaleuritate films, we have used a melt-polycondensation polymerization in air at different times (8, 12, 16, and 22 h) and temperatures (150, 170, 190, and 200°C). Special emphasis has been paid to the effect of oxygen in the structure, composition, and physical (i.e., thermal, mechanical, and optical) properties of synthesized polyaleuritates as well as the use of Ti(OiPr)_4_ as catalyst.

## Experimental

Aleuritic (9,10,16-trihydroxyhexadecanoic) acid was purchased from Alfa Aesar (purity ≥ 95%) and used as received.

Polyaleuritate films were prepared by direct melting of aleuritic acid in a pre-heated (~110°C) open carbon doped Teflon mold and further heating for 8, 12, 16, and 22 h at 150, 170, 190, and 200°C. For catalyzed samples, few drops of a Ti(OiPr)_4_ solution in *n*-butanol was previously added to the acid and stirred to obtain a slurry. Final catalyst content was 400 ppm. The solvent was removed under vacuum for 3 days. In both series, obtained film thickness was about 350 μm.

FTIR spectrometer was a Nicolet IS50 working at 4 cm^−1^ resolution. 32 and 50 scans were accumulated for transmission and attenuated total reflection (ATR), respectively. Processing and band fitting was performed with the Nicolet Omnic software. ATR spectra were corrected to account for the depth dependence of the signal.

Solid-state ^13^C NMR spectra were acquired with a Bruker Avance III HD 400 MHz operating in direct polarization at magic-angle. A 30° ^13^C pulse of 1.2 μs and a delay time of 40 s was used and 300 scans were accumulated. ^13^C-chemical shifts were referred to tetramethylsilane.

Differential Scanning Calorimetry (DSC) thermograms were acquired with a DSC Q20 (TA Instruments) from −70 to 150°C under nitrogen flow (50 mL/min) at 10°C/min using non-hermetic aluminum pans and about 4 mg of sample. *T*_g_ were determined from the second heating once adsorbed water was removed. Thermal Gravimetric Analysis (TGA) was carried out with a SDT Q600 TGA/DSC analyzer (TA Instruments). The samples (~8 mg) were heated from RT to 500°C at 5°C/min with N_2_ flowing at 100 mL/min. In addition to T_d5%_, T_donset_ values have been obtained from the slope change in the TGA profile (see Figure S1 in Supporting Information).

An X'Pert Pro (PANalytical) diffractometer with monochromated CuK_α_ radiation was used for Wide-Angle X-ray Diffraction (WAXD) analysis. Diffractograms were collected at 45 kV, 40 mA between 3 and 70° (2θ) in 0.0501° steps. The films were placed on a zero background silicon single crystal plate in a θ−2θ configuration. Crystalline indexes are calculated from the equation

(1) $$\begin{array}{r} CI\;=\;\frac{100\;\times Ac}{Ac+Aa} \end{array}$$

where *Ac* and *Aa* are the areas of the crystalline and amorphous peaks.

Tensile tests were performed with a MTS Criterion 42 machine equipped with a 50 N load cell and applying a 0.02 N preload. Rectangular uniform pieces (7 × 30 mm) were brought to rupture at a constant deformation rate of 5 mm/min at room conditions. Mechanical parameters were calculated using the specimen cross-section under no applied load and averaged from at least 6 samples for each preparation. A Q-800 DMA (TA Instruments) was used for creep-recovery and strain relaxation measurements. Pieces of about 20 × 7 mm were subjected to 0.2 MPa stress and 0.5% deformation, respectively. A CSM UNHT ultrananoindenter equipped with a Berkovich pyramidal tip was used for nanoidentation experiments. All measurements were done with 30 s loading and unloading time. Young's modulus and hardness were calculated from the unloading curves with the Oliver & Pharr method, assuming a Poisson ratio ν = 0.3.

Atomic Force Microscopy images were obtained with a Nanotec AFM in low amplitude dynamic mode and using Nanosensors PPP-NCH levers (295 kHz resonance frequency, 29 N/m force constant).

Static Water Contact Angle (WCA) values were determined with an Attension TL100 Optical Tensiometer by the sessile drop method and image profile analysis and using a 3 μL Milli-Q grade water drop.

Water vapor permeability was measured using methacrylate cups, with the polyester film sealing a partially filled water reservoir, and placed inside a dry chamber (RH <1%). Water transmission rate (WVTR) was obtained from the slope of the weight loss vs. time plot and referred to the permeation area. Values were normalized for 25 μm thickness by the factor: film thickness (μm)/25.

UV-visible curves were obtained by using a Cary 300 spectrometer equipped with a barium sulfate coated integrating sphere (Labsphere).

## Results and Discussion

### Chemical Characterization

The polymers synthesized in this work were insoluble in solvents like light alcohols and chloroform. Only the one prepared in the mildest conditions was partially dissolved (6% w/w) in chloroform after several days soaking at room temperature. For this reason, solid-state chemical characterization techniques such as ATR-FTIR and Direct Polarization/Magic Angle Spinning (DP/MAS) ^13^C MAS were used.

**Figure 1:**
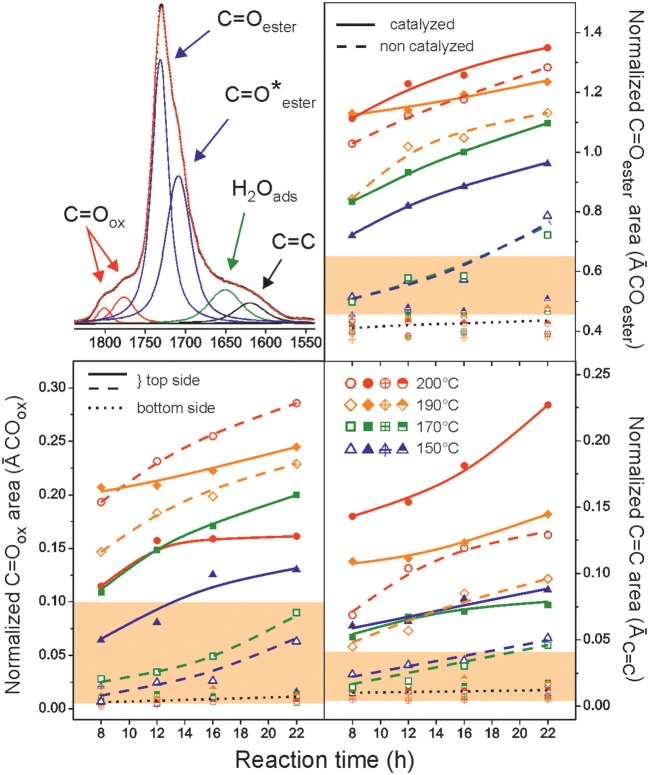
ATR-FTIR band area evolution (normalized to the area of the methylene group stretching) of detected species in polyaleuritates films prepared in air as a function of time and reaction temperature. Data for the air exposed (top) and air preserved (bottom) sides are shown. The filled regions (light red areas) correspond to the value range obtained from the corresponding bulk transmission IR spectra of both (catalyzed and non-catalyzed) series. In the upper left corner the ATR spectrum of the air-exposed side of the polymer in the 1,825–1,540 cm^−1^ region and its main contributors are included. Asterisk denotes crystalline phase and/or hydrogen bond ester (C=O) groups.

The polyaleuritates prepared in this work were characterized by ATR-FTIR spectroscopy as polyhydroxylated fatty esters with characteristic ν(C=O) at 1,735 cm^−1^, ν(C-O-C) bands at 1,248 and 1,177 cm^−1^, and ν(C-O), and ν(O-H) of hydroxyls in the 1,050–1,110 cm^−1^ and 3,550–3,250 cm^−1^ regions, respectively, as well as intense ν(–CH_2_-) peaks around 2,930 and 2,854 cm^−1^ (Bellamy, 1975; Benítez et al., 2015c) (wider range spectra are available in Figures S2–S5 in Supporting Information). The absence of free acid peaks around 1,700 cm^−1^ is indicative of a high esterification degree. The ν(C=O) region of polyaleuritates has been studied in detail and several contributions were distinguished: (i) the peak at 1,735 cm^−1^ assigned to ester carbonyls in the amorphous phase, (ii) the shoulder at 1,715 cm^−1^ attributed to crystalline phase and/or hydrogen bonded ester (C=O) groups (Xu et al., 2002; Honma et al., 2003; Kansiz et al., 2007), (iii) the band at 1,645 cm^−1^ mostly caused by adsorbed water molecules, (iv) the absorption at 1,615 cm^−1^ ascribed to dehydration (-C=C-) by-products, and (v) 1,803 and 1,773 cm^−1^ weak bands associated with oxidized species such as peroxyesters (R-(CO)-O-O-R′) and diacyl peroxides (R-(CO)-O-O-(CO)-R′) (Davison, 1951). ATR spectra were fitted to these components and their evolution (as normalized to the area of the ν(-CH_2_-) peaks) with temperature and reaction time is shown in Figure 1. An intense and progressive increase of ester bonds and oxidation and dehydration by-products as the reaction conditions become harsher was observed. Such a behavior was related to the presence of oxygen in the reaction atmosphere because no modifications were observed at the side facing the mold (bottom side) (Figure S3 in Supporting Information).

From ATR data, the Ti(OiPr)_4_ catalyst favored the secondary esterification as well as the oxidation and dehydration side processes. However, the presence of oxidized species decreased at higher temperature, suggesting that the catalyst might also facilitate their thermal decomposition.

ATR-FTIR is an analytical technique with a limited penetration depth of about 1–5 μm in solids. Though it is very suitable to detect near-surface chemical modifications, it may not provide reliable bulk information. To evaluate the magnitude of the oxidation and dehydration processes within the films, transmission IR spectra were also measured (the carbonyl region is available in Figure S6 in Supporting Information). The same species as in ATR-FTIR were detected in both series, but their relative intensity values (filled regions in light red color in Figure 1) were more uniform and much lower. These transmission IR data revealed that the chemical alteration was quite limited to the air-exposed side and the bulk remained essentially as an intact polyester.

**Figure 2:**
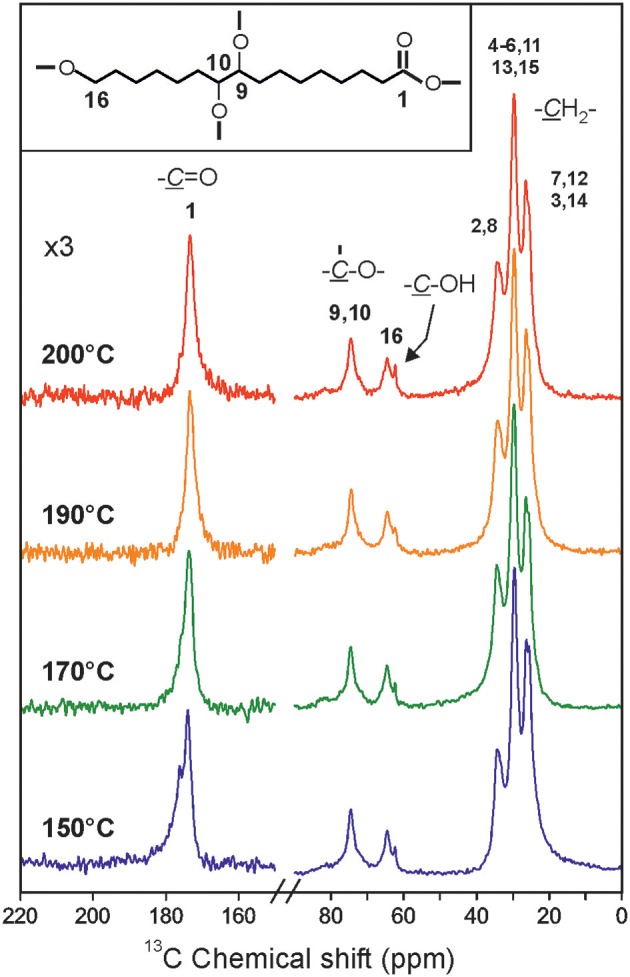
^13^C solid-state NMR spectra of polyaleuritate prepared by melt-polycondensation in air at the indicated temperature for 22 h.

To further confirm the bulk chemical composition of polyaleuritates, solid-state ^13^C NMR spectra were collected. Figure 2 shows the corresponding spectra of the polymers polymerized during 22 h at 150, 170, 190, and 200°C. Despite the differences in the reaction temperature, almost no variation was observed. In all cases, the high esterification degree was confirmed by the characteristic (C=O) peak in esters at 174 ppm (Zlotnik-Mazori and Stark, 1988; Ahmed et al., 2003; Deshmukh et al., 2003). Particular interest was paid to check the absence of free acid (~177 ppm), which was confirmed from the lack of the ~8 ppm signal in the ^1^H spectra (not shown). The esterification was mostly with the primary hydroxyl (~80%), as revealed by the intense peak at 65 ppm, with little presence of unreacted groups at 62.5 ppm (Benítez et al., 2015b). Chemical changes revealed by ATR-FTIR on the air exposed side, i.e., the presence of oxidation by-products such as peroxyesters and peroxides and the increment of ester bonds, were not detected from ^13^C NMR peak shape and intensity modifications (Benítez et al., 2017). Consequently, NMR data supported the low degree of bulk alteration of the synthesized polyaleuritates and its restriction to the near-surface region on the films.

**Scheme 1:**
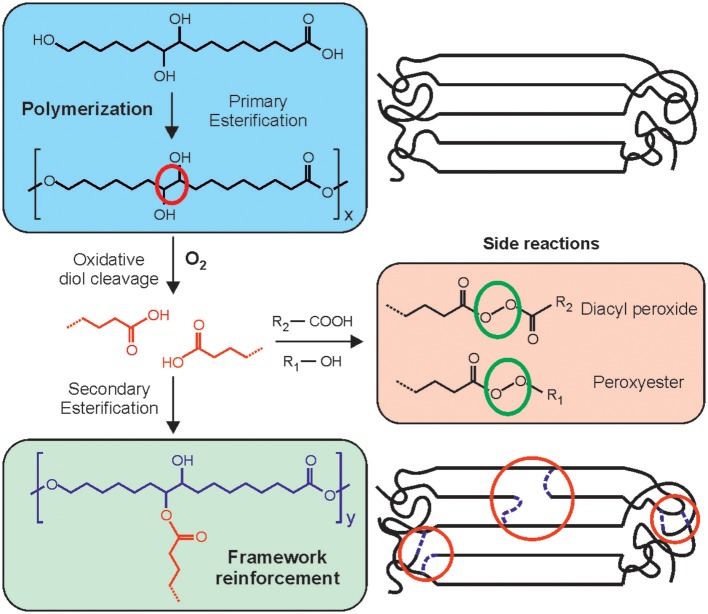
Proposed reaction mechanism for the melt-polycondensation of aleuritic acid in air. The effect of the diol cleavage and secondary esterification in both the crystalline and the amorphous domains of polyaleuritates are depicted.

Based on these findings, a melt-polycondensation mechanism in which the polyaleuritate network is essentially made by ester bonds involving the primary hydroxyls was proposed, Scheme 1. In the presence of O_2_, the diol moiety suffers an oxidative cleavage to yield two additional carboxylic acid groups that can subsequently undergo a secondary esterification with surplus secondary hydroxyls. The result is a bond densification due to the creation of two new ester linkages per each cleaved C-C bond. Side oxidation of either or both ester and –COOH groups leads to the formation of peroxyesters and diacyl peroxides.

### Thermal Properties

**Figure 3:**
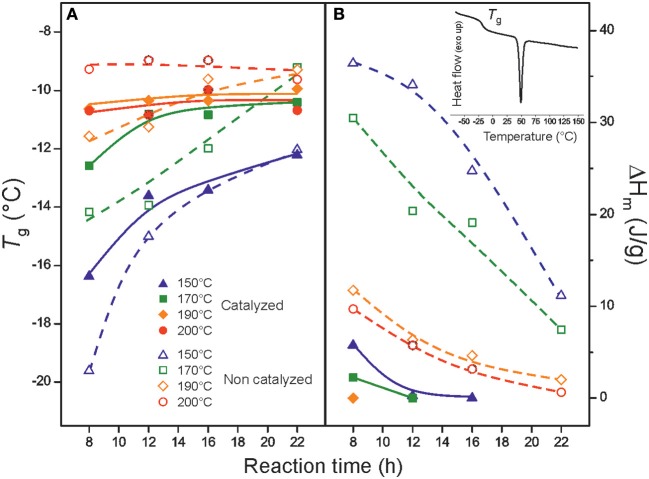
**(A)** *T*_g_ and **(B)** melting enthalpy (ΔH_m_) values of polyaleuritates as a function of temperature and reaction time for both catalyzed (filled symbols) and non-catalyzed (open symbols) series. The inset in **(B)** shows the DSC thermogram of the polyaleritate synthesized at 170°C during 8 h with a low temperature glass-transition and a melting peak ~50°C as a representative example.

The DSC thermograms of the synthesized polyaleuritates displayed a glass transition between −20 and −9°C and, those prepared at milder conditions, a melting ~50°C. The intense glass transition was an indication of low crystallinity in these polyesters. *T*_g_ values depended on the preparation conditions and were increased as temperature and reaction time were raised until stabilization around −9°C, Figure 3A. Such an increase can be envisaged as a hindrance of chain mobility due to branching caused by partial esterification of secondary hydroxyls and the associated growth of molecular weight (Benítez et al., 2015b). In general, these glass transition temperatures were lower than those of poly(lactic acid) (40–70°C) and polyethylene terephthalate (73–80°C), higher than the *T*_*g*_ of polycaprolactone (~ −60°C), and in the same range than polyhydroxyalkanoates (from −30 to 10°C), plant-derived aliphatic polyhydroxyesters (from −44 to −5°C), and tomato pomace-polyesters (from −30 to −5°C) (Heredia-Guerrero et al., 2019). On the other hand, the melting peak was associated with crystalline domains of the linear primary ester structure and quickly decreased as the reaction conditions become harsher, Figure 3B. The increase of melt viscosity caused by branching and the presence of oxidized ester groups may restrict chain mobility and hampers the packing into a crystalline structure.

Reaction time needed to reach *T*_g_ stabilization and infusibility depend on the esterification kinetics within the melt and was found to be sensitive to impurities in the commercial aleuritic acid used (extracted from the natural resin shellac). In general, *T*_g_ values are slightly higher and stabilization time is reduced as the aleuritic acid purity is lower (Benítez et al., 2015c). This is very likely caused by residual doubly functionalized organic molecules acting as cross-linkers and accelerating the coalescence of domains by reaction with surplus secondary hydroxyls.

*T*_*g*_ stabilization and melting enthalpy reduction were faster in the catalyzed series. This is interpreted as the participation of the Ti(OiPr)_4_ in either or both the secondary hydroxyl esterification or the diol cleavage reaction, as suggested by the more intense increment of ester bonds observed by ATR-FTIR.

Regarding the thermal stability of polyaleuritates, no meaningful differences were observed in TGA curves independently of the preparation conditions used (see Figure S7 in Supporting Information). Two concatenated decomposition stages around 408 and 455°C representing the 95% weight loss were observed. Additionally, the characteristic weight loss of aleuritic acid around 235°C was not detected, supporting IR and NMR data about the achievement of a full conversion. TGA parameters are compiled in Table 1. As observed, the maximum decomposition temperature (T_dmax_) was constant within the series. T_d5%_ and T_donset_ values were also quite stable, but 11–13°C lower for the catalyzed samples. Such a temperature shift was tentatively associated with a residual activity of the embedded catalysts toward some kind of thermal decomposition (oxidized species, for instance).

**Table 1:** T_d5%_, T_donset_, and T_dmax_ values obtained from the thermal decomposition of polyaleuritates prepared for 22 h at the indicated temperatures.

**Reaction temperature (°C)**	**T**_******d5%******_		**T**_******donset******_		**T**_******dmax******_	
	**Non-catalyzed**	**Catalyzed**	**Non-catalyzed**	**Catalyzed**	**Non-catalyzed**	**Catalyzed**
150	327	316	353	345	411	412
170	341	319	362	348	409	407
190	333	318	357	344	409	407
200	325	321	353	343	409	407

### Crystallinity of Polyaleuritates

**Figure 4:**
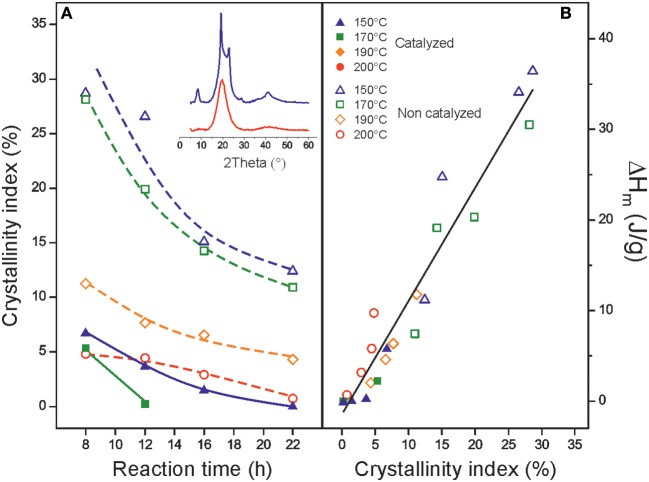
**(A)** Crystallinity values calculated from XRD data of polyaleuritates as a function of the reaction time. The inset shows XRD patterns for samples prepared in air at 150°C (blue) and 200°C (red) for 8 h. **(B)** Relationship between the melting enthalpy values and the XRD calculated crystallinity indexes.

Wide-Angle X-Ray (WAXD) diffractograms of polyaleuritates (Figure 4A, inset) showed weak peaks at 2θ = 19.25, 22,81, 28.67, 36.28, and 41.26° with d-spacing of 4.61, 3.88, 3.11, 2.47, and 2.19 Å, respectively, assigned to the (110), (200), (210), (020), and (310) planes of an orthorhombic packing of methylene fragments in linear fatty polyesters (Fuller and Frosch, 1939; Menges et al., 2007). However, diffractograms were dominated by a broad halo centered around 19.50° that corresponds to the amorphous phase indicative of a low crystallinity. CI values were below 30% and systematically decreased as the temperature and reaction time was raised, Figure 4A. Several factors can be proposed for such amorphization, among them, the steric effect of secondary hydroxyls and/or chain branching caused by partial esterification of such hydroxyls. In this sense, the CI value of the polyester synthesized in vacuum from 16-hydroxyhexadecanoic acid (the analogous C_16_ ω-hydroxy acid having no secondary hydroxyls) is ~80 vs. the ~35% for polyaleuritate prepared in the same inert conditions (Benítez et al., 2015a). Amorphization was also caused by the presence of oxygen in the reaction atmosphere. It has been previously observed in the polymerization of the 16-hydroxyhexadecanoic acid and attributed to the lattice mismatch caused by the generation of peroxyester groups (Benítez et al., 2017). In the case of polyaleuritate, the oxidative diol cleavage was an additional mechanism for secondary hydroxyl esterification and branching. Again, the lower crystallinity of polyaleuritate prepared in air with respect to the polyester of 16-hydroxyhexadecanoic acid (where no diol cleavage is possible) is a support for such argumentation.

The catalytic activity of Ti(OiPr)_4_ toward the oxidative cleavage and the secondary esterification justifies the acceleration of the amorphization process in the catalyzed series, Figure 4A. With catalysis, amorphous polyesters were readily obtained at moderated temperature and reaction time.

In Figure 4B, the calculated CI and the melting enthalpy (ΔH_m_) of both series were linearly correlated. The trend observed predicts a melting enthalpy of 125 ± 8 J/g for a hypothetical fully crystalline polyaleuritate. Such value is above the one for PLA (93 J/g) (Garlotta, 2001) and comparable to PCL (167 J/g) (Chen et al., 1998).

### Mechanical Characterization

**Figure 5:**
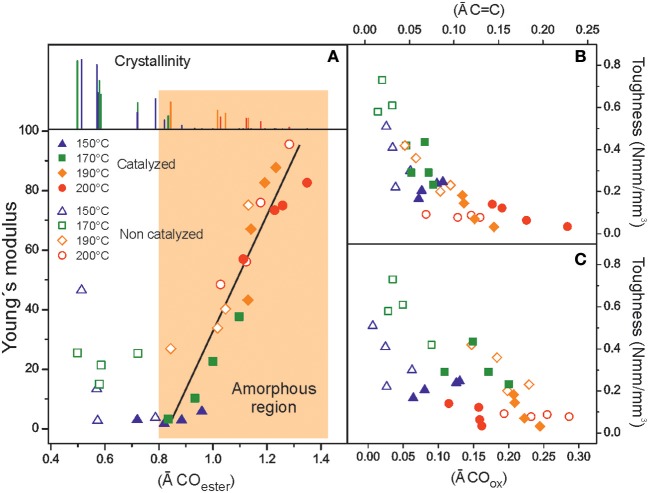
**(A)** Relationship between the elastic modulus and the intensity of the ester carbonyl ATR band in polyaleuritates synthesized in air. The dependence is well-defined for amorphous samples (light orange area). Tensile toughness variation with the presence of **(B)** dehydration, and **(C)** oxidation by-products.

The mechanical behavior of polyaleuritate films was studied by uniaxial tensile tests. The calculated parameters are compiled in Table 2. Stress-strain curves (see Figure S8 in Supporting Information) showed a ductile pattern with uniform extension (no necking) up to ~50% elongation. As the reaction time and temperature were increased, the viscoelastic region was reduced and the polyester became brittle with low rupture strain values (ε_rupture_ ~3–4%) For the catalyzed series, the ductile to brittle transition was reached at milder reactions conditions. The Young's modulus (E) of polyaleuritates fell to its minimum (2–3 MPa) at 150°C. This value is characteristic of very soft rubbery materials and can be ascribed to the amorphization process. Above such preparation temperature, the stiffness increased in both series up to 90–95 MPa and the rupture stress peaked around 4 MPa. Residual crystallinity strongly affects the mechanical parameters of polyaleuritates prepared at the mildest conditions. In the rest of the series, samples are highly amorphous and the elastic modulus can be linearly correlated with the ATR-FTIR normalized area of ester groups, Figure 5A. The increase of rigidity was then associated with the densification of ester bonds in the polyaleuritate framework as a consequence of the secondary esterification after the oxidative diol cleavage.

**Table 2:** Tensile parameters for polyaleuritates prepared by melt-polycondensation in air.

	**Non-catalyzed**				**Catalyzed**			
**Reaction time (h)**	**E (MPa)**	**σ_rupture_ (Mpa)**	**ε_rupture_ (%)**	**Toughness (Mpa)**	**E (Mpa)**	**σ_rupture_ (Mpa)**	**ε_rupture_ (%)**	**Toughness (Mpa)**
	150°C							
8	47 ± 6	3.5 ± 0.6	17 ± 7	0.5 ± 0.1	3 ± 0.7	0.6 ± 0.3	42 ± 9	0.13 ± 0.08
12	13 ± 2	1.1 ± 0.5	31 ± 9	0.4 ± 0.1	1.7 ± 0.2	0.7 ± 0.1	52 ± 5	0.20 ± 0.06
16	2.7 ± 0.3	0.6 ± 0.1	35 ± 8	0.2 ± 0.1	2.9 ± 0.6	1.2 ± 0.2	46 ± 6	0.24 ± 0.07
22	3.7 ± 0.9	1.2 ± 0.5	40 ± 8	0.3 ± 0.1	5.7 ± 0.3	1.5 ± 0.3	30 ± 7	0.25 ± 0.09
	170°C							
8	25 ± 7	2.5 ± 0.5	27 ± 9	0.6 ± 0.2	3.2 ± 0.9	1.1 ± 0.1	47 ± 9	0.3 ± 0.1
12	15 ± 1	3.1 ± 0.7	39 ± 5	0.7 ± 0.3	10.4 ± 0.7	1.7 ± 0.3	38 ± 8	0.4 ± 0.1
16	21 ± 5	2.6 ± 0.6	31 ± 4	0.6 ± 0.3	23 ± 5	2.0 ± 0.6	21 ± 9	0.3 ± 0.1
22	25 ± 1	2.5 ± 0.4	23 ± 3	0.4 ± 0.1	38 ± 8	2.5 ± 0.4	11 ± 6	0.2 ± 0.1
	190°C							
8	27 ± 5	2.2 ± 0.7	27 ± 7	0.4 ± 0.2	43 ± 7	2.0 ± 0.4	10 ± 6	0.18 ± 0.07
12	34 ± 3	2.6 ± 0.6	23 ± 9	0.4 ± 0.1	67 ± 9	3.5 ± 0.6	7 ± 3	0.14 ± 0.09
16	40 ± 5	2.8 ± 0.4	15 ± 6	0.2 ± 0.1	83 ± 10	2.8 ± 0.9	5 ± 3	0.07 ± 0.06
22	75 ± 5	3.4 ± 0.8	10 ± 3	0.3 ± 0.1	88 ± 12	1.8 ± 0.7	3 ± 1	0.03 ± 0.02
	200°C							
8	48 ± 4	2.4 ± 0.6	8 ± 3	0.09 ± 0.08	57 ± 9	3.5 ± 0.9	9 ± 3	0.14 ± 0.08
12	56 ± 8	2.7 ± 0.2	6 ± 2	0.08 ± 0.04	73 ± 12	4.1 ± 0.9	6 ± 2	0.12 ± 0.09
16	76 ± 7	2.6 ± 0.6	4 ± 2	0.09 ± 0.06	75 ± 5	3.6 ± 0.8	4 ± 2	0.07 ± 0.06
22	96 ± 10	3.7 ± 0.9	3.5 ± 0.9	0.08 ± 0.07	76 ± 7	2.5 ± 0.7	4 ± 1	0.04 + 0.03

**Figure 6:**
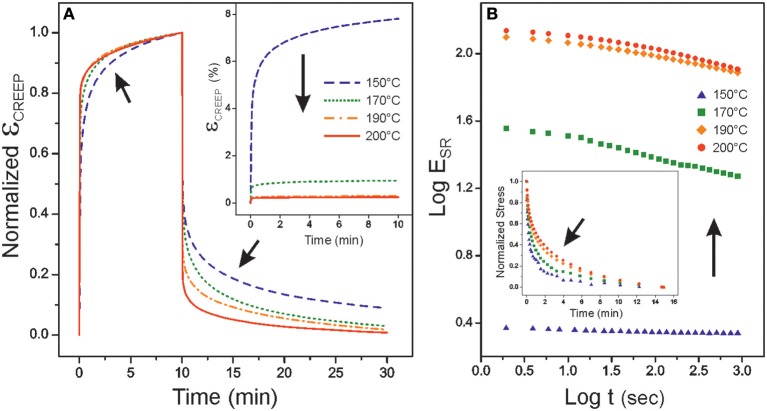
**(A)** creep-recovery (σ = 0.2 MPa) and **(B)** strain relaxation (ε = 0.5%) curves of polyaleuritate films prepared in the 150–200°C range for 22 h in air. Arrows are included to highlight results indicating a structural reinforcement.

The structural reinforcement was confirmed by creep and strain relaxation measurements. The time response of polymers under stress is widely used to extract valuable information about their structure. For instance, in creep-recovery experiments, robust crosslinked frameworks are characterized by a low strain, a flat equilibrium region and a fast recovery due to the absence of flow under stress (Macosko, 1994; Menard, 2008). Creep and stress relaxation performance is affected by other intrinsic factors such as crystallinity and whether measurements are carried out above or below the glass transition temperature. In the series, *T*_g_ values were below room temperature. Hence, all samples were in the rubbery state and, for the tests, low crystallinity specimens were selected. Creep-recovery curves of polyaleuritate synthesized from 150 to 200°C for 22 h are plotted in Figure 6A. The inset shows the strong reduction of strain (ε) as the polymerization temperature was raised while the normalized creep-recovery data (main body) revealed a progressive flattening of the equilibrium region and a faster recovery. Such a behavior can be related to a structural reinforcement and a decrease of flow as the preparation temperature of polyaleuritate was increased. Similarly, stress relaxation experiments (Figure 6B) showed higher relaxation moduli (E_SR_) and slower stress decays (inset), as expected from the increment of stiffness and the reduction of flow as the reaction temperature was higher.

It is interesting to notice that the mechanical behavior of these polyesters seems to reach a steady state at higher preparation times and temperatures. This observation is the confirmation that the diol cleavage and secondary esterification is a limited process, restricted to the nearby regions of the polymer-air interphase and not progressing into the entire bulk of the film. Most likely, oxidation is a self-regulated process as the viscosity of the melt phase increases upon densification of ester bonding and hampers further oxygen diffusion into the bulk.

The toughness of polyaleuritates was also evaluated by calculating the area under the stress-strain curve up to the breaking point. In general, there was an increase in the specific mechanical energy needed for rupture up to the 170°C series, Table 2. However, after that, toughness decreased significantly when harsher preparation conditions were used. Such a reduction seems contradictory with the proposed structure reinforcement under oxidative conditions. However, toughness can be inversely related with the presence of oxidation and dehydration by-products, Figures 5B,C. In this sense, it is postulated that the altered air-exposed side of the film becomes a stress concentration region where cracks are more easily created and propagated to the bulk leading to rupture at lower strain.

**Figure 7:**
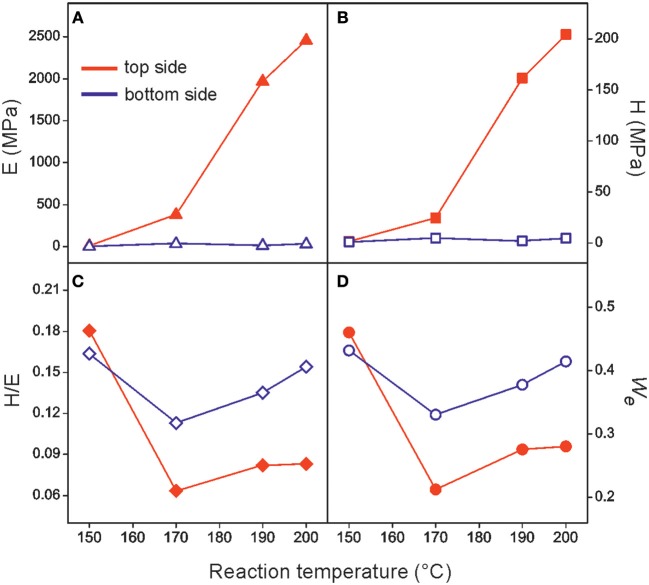
Indentation parameters: **(A)** E, elastic modulus, **(B)** H, hardness, **(C)** H/E, elastic strain to failure, and **(D)** *W*_*e*_, elastic recovery for the air exposed (top) and the air preserved (bottom) sides of polyaleuritate films synthesized in air for 22 h at the indicated temperatures.

To address this issue, the local mechanical properties of both the air exposed (top) and the air preserved (bottom) sides were characterized by nanoindentation, Figure 7. Both layers were tested at several loads in the 300–5,000 μN range. While no appreciable differences were found at the bottom side, the oxidized region was depth-dependent. In this case, the lowest load was used to minimize the influence of the underlying bulk. Elastic modulus (E) and hardness (H) values for both sides are plotted as a function of the temperature for the 22 h non-catalyzed series in Figures 7A,B, respectively. As observed, there were no meaningful differences between both sides in the sample prepared at 150°C, but, in the rest of the series, both parameters were much higher (up to two orders of magnitude) for the air exposed surface. Then, oxidation led to a stiffer and harder material in agreement with tensile results. Related to toughness and resistance to fracture, the (H/E) ratio is defined as the elastic strain to failure (Leyland and Matthews, 2000) and the elastic recovery (*W*_*e*_) is a parameter associated with the energy dissipation through deformation (Recco et al., 2009). As reported in Figures 7C,D, top-side (H/E) and *W*_*e*_ values were lower than bottom-side ones. This is interpreted as a lower resistance to accommodate mechanical stress and to dissipate energy through deformation and, therefore, as a higher susceptibility to fracture of the oxidized side of the film. Indentation results support the hypothesis about the initiation of fracture at this altered layer and the toughness reduction observed in tensile tests.

### Surface Texture and Water and UV-Vis Light Barrier Properties

**Figure 8:**
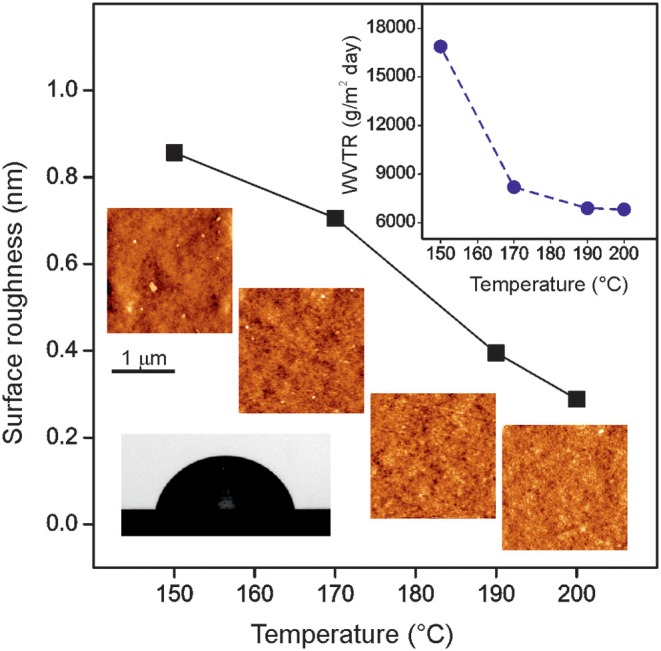
AFM topographic images and surface RMS roughness values of the air exposed side of polyaleuritates synthesized in air for 22 h at the indicated temperature. The upper inset shows the normalized water vapor transmission rate of these samples at 25°C. The lower inset displays the profile of a water drop on top of the air exposed side of a polyaleuritate film.

If intended to be used as food preserving films and coatings, the permeability of polyaleuritates to water and UV-vis radiation is an essential issue to be addressed. Since the texture of the air exposed side of films was too smooth to be resolved by scanning electron microscopy (SEM) (see Figure S9 in Supporting Information) and atomic force microscopy (AFM) was used. Topographic AFM images showed that the increase of reaction conditions leads to a noticeable reduction of surface roughness, Figure 8. In parallel, the water vapor transmission rate (WVTR) also decreased by a factor of two in this series (inset in Figure 8). Likely, the textural surface reduction is the factor conditioning the water permeability by the diminishment of the effective cross section for the diffusion process. WVTR values of polyaleuritates are about 40 times higher than those of other biodegradable polyesters such as PLA and PCL, but comparable to cellulose acetate (Shogren, 1997). Wettability of polyaleuritates was moderate, with WCA values around 74°C, slightly below those of PCA and PCL, around 85° and 97°, respectively (Pinto et al., 2013; Wang et al., 2017). The presence of polar free hydroxyls in the polyester framework due to the 3:1 OH:COOH ratio in the monomer was responsible for such a moderate hydrophobicity and water barrier properties. Indeed, the introduction of polar groups in PLA and PCL causes a WCA reduction up to 15–20° (Janorkar et al., 2004; Wang et al., 2017) and the value for the polyester obtained from 16-hydroxyhexadecanoic acid (with a 1:1 OH:COOH ratio) is around 83°.

**Figure 9:**
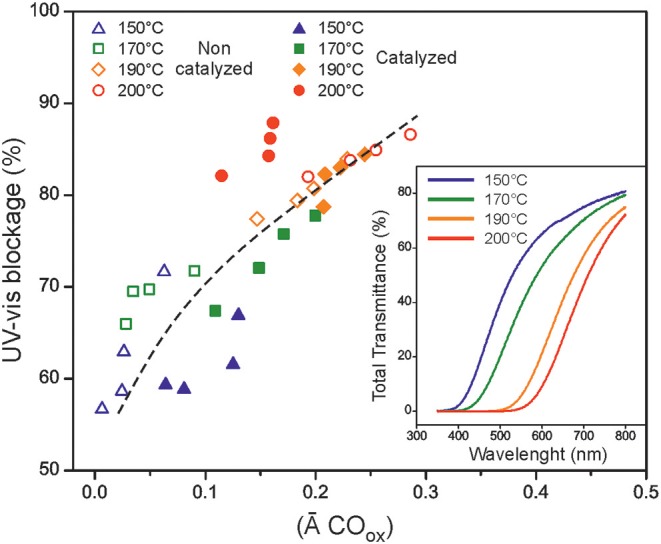
Relationship between the UV-vis blockage and the presence of oxidized species at the air exposed side of films. The inset shows the total transmittance UV-vis curves of polyaleuritates prepared in air for 16 h at the indicated temperatures.

Films prepared in air by melt-polycondensation progressively colors from pale yellow to brown with transparency values (total transmittance at 600 nm) ranging from 74 to 6% as the temperature and the reaction time are increased, Table 3. However, synthesized polyaleuritates were quite effective in blocking the UV radiation. They show a 0% transmittance plateau continuously growing from (200 to 350 nm) up to (200 to 540 nm) along the series, Figure 9 (inset). To evaluate the overall UV-vis blocking capacity, the area above the transmittance curve was calculated in the 200–800 nm region. As observed in Figure 9, blockage values were quite high (56 to 88%) and can be related to the presence of oxidized species at the air exposed side of the films. Such species were responsible for the coloring effect and contribute to the absorption component of the non-transmitted radiation. The deviation observed for the catalyzed series at 200°C was due to a low degree of carbonization that increased the overall absorption. The slightly higher blockage of non-catalyzed samples obtained at mild conditions is due to their higher crystallinity and to the enhancement of the reflected component.

**Table 3:** Transparency values (as the total transmittance at 600 nm) for polyaleuritate films as a function of the preparation conditions.

**Transparency (%)**	**Time (h)**			
**Temperature (°C)**	**8**	**12**	**16**	**22**
150	74	72	65	49
170	61	53	54	48
190	31	25	21	13
200	17	13	9	6

## Conclusions

The preparation of insoluble and infusible polyhydroxyester films from a natural occurring polyhydroxylated fatty acid (aleuritic acid) by direct polycondensation in melt state has been described. The method is simple, uses no hazardous substances and becomes of particular interest for the preparation of innocuous thermostable and solvent resistant materials from renewable sources. Compared to soluble thermoplastic short chain polyhydroxyalkanoates (PHAs) and PLA, polyaleuritates prepared in air are adequate materials to resist thermal sterilization procedures and the migration to foodstuff when used in food packaging.

The absence of free acid chemical fingerprints reveals a high reaction conversion. The polyester formed is preferentially with the primary –OH group leaving a considerable population of free secondary vicinal hydroxyls. The presence of O_2_ in the reaction atmosphere induces an oxidative diol cleavage reaction and the formation of new acid groups that further undergo a secondary esterification stage with surplus hydroxyls. The result is a densification of the ester linkage network gradually fading from the air exposed side toward the interior of the film that noticeably affects the bulk structural arrangement and mechanical performances. Thus, as reaction time and temperature are raised, a progressive amorphization and stiffening of polyaleuritates is observed. Amorphization is caused by the increment of branching due to esterification of secondary hydroxyls and the derived disruption of the packing of linear chains. Ester bond densification is also postulated for the surface compaction and the reduction of the water transmission rate of polyaleuritate films. Beside the collateral increment of ester bonding, the participation of oxygen causes a series of side reaction giving rise to peroxy compounds. Such species color the films and improve their UV-vis blocking capacity, which is an important issue if envisaged as a food protecting material. Though such chemical and structural alterations are limited to few microns depths at the air-exposed side of the films, it is of relevance as the thickness of many coatings used in food packaging is similar.

The addition of Ti(OiPr)_4_ as a catalysts has a mild activity because of diffusion problems within the melt but it is observed to have a positive effect in both the secondary esterification and the formation of oxidized species, particularly at low and moderate reaction temperatures. In any case, there is not a significant reduction in temperature or reaction time to justify its use.

In view of the results, these polyaleuritates show interesting features as food packaging materials and potentiates the valorization of other fatty polyhydroxyacids from renewable cheap agricultural wastes such as fruit pomaces.

## Data Availability Statement

All datasets generated for this study are included in the manuscript/Supplementary Files.

## Author Contributions

Experimental work was performed by JB with the supervision of AH and contributions from JH-G (ATR-FTIR), SG-P (WCA, permeability), MC-C (IR transmission, Tensile), LC (nanoindentation), AG (UV-vis). All authors equally participated in the analysis and discussion of results and the writing of the paper.

### Conflict of Interest

The authors declare that the research was conducted in the absence of any commercial or financial relationships that could be construed as a potential conflict of interest.
