# Supplementary material for: Insoluble and Thermostable Polyhydroxyesters From a Renewable Natural Occurring Polyhydroxylated Fatty Acid
Source: Front Chem. 2019 Sep 24;7:643. doi: 10.3389/fchem.2019.00643 (PMC6768952; doi:10.3389/fchem.2019.00643)
Supplement: Supplementary file 1 [file Table_1.DOCX]

**Insoluble and thermostable polyhydroxyesters from a renewable natural occurring polyhydroxylated fatty acid**

*José J. Benítez^1*^, Susana Guzman-Puyol^2,5^, Miguel A. Cruz-Carrillo^3^, Luca Ceseracciu^4^, Ana González Moreno^2^, Antonio Heredia^2,5^, and José A. Heredia-Guerrero^2,5*^*

^1^ Instituto de Ciencia de Materiales de Sevilla (ICMS). Centro Mixto CSIC-Universidad de Sevilla. Americo Vespucio 49. Isla de la Cartuja. 41092-Seville (Spain).

^2^ IHSM La Mayora, Departamento de Biología Molecular y Bioquímica. Universidad de Málaga. 29071-Málaga (Spain).

^3^ Facultad de Ingeniería Culiacán, Universidad Autónoma de Sinaloa, Blvd. de las Américas s/n, 80040-Culiacán Rosales, Sinaloa (Mexico).

^4^ Materials Characterization Facility. Istituto Italiano di Tecnologia (IIT). 16163-Genova (Italy).

^5^ Instituto de Hortofruticultura Subtropical y Mediterránea (IHSM) La Mayora. Universidad de Málaga-CSIC. 29750-Málaga (Spain).

**Supporting information**

**Figure S1.** Definition of the onset point from the TGA thermogram. In addition to the Td 5% temperature, this parameter is considered as an indicator for the thermal stability of polyaleuritates obtained.


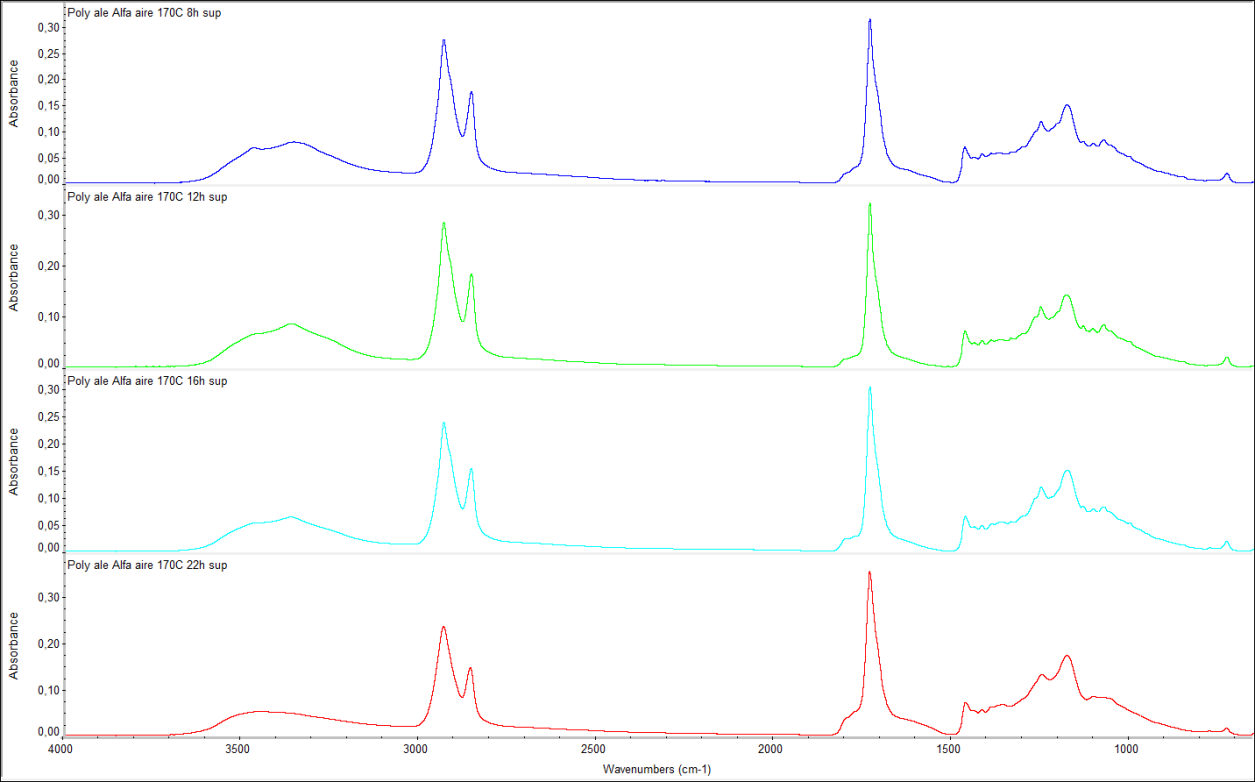


**Figure S2.** Full range ATR-FTIR spectra of the air exposed side of polyaleuritate films prepared at 170°C with no catalyst. The formation of the ester is confirmed by characteristic peaks at 1735 cm^-1^ (ν(C=O)) and 1248 and 1177 cm^-1^ (ν(C-O-C)). A clear increment in the ν(C=O)/ν(CH_2_) band area ratio is observed as the reaction time is increased.


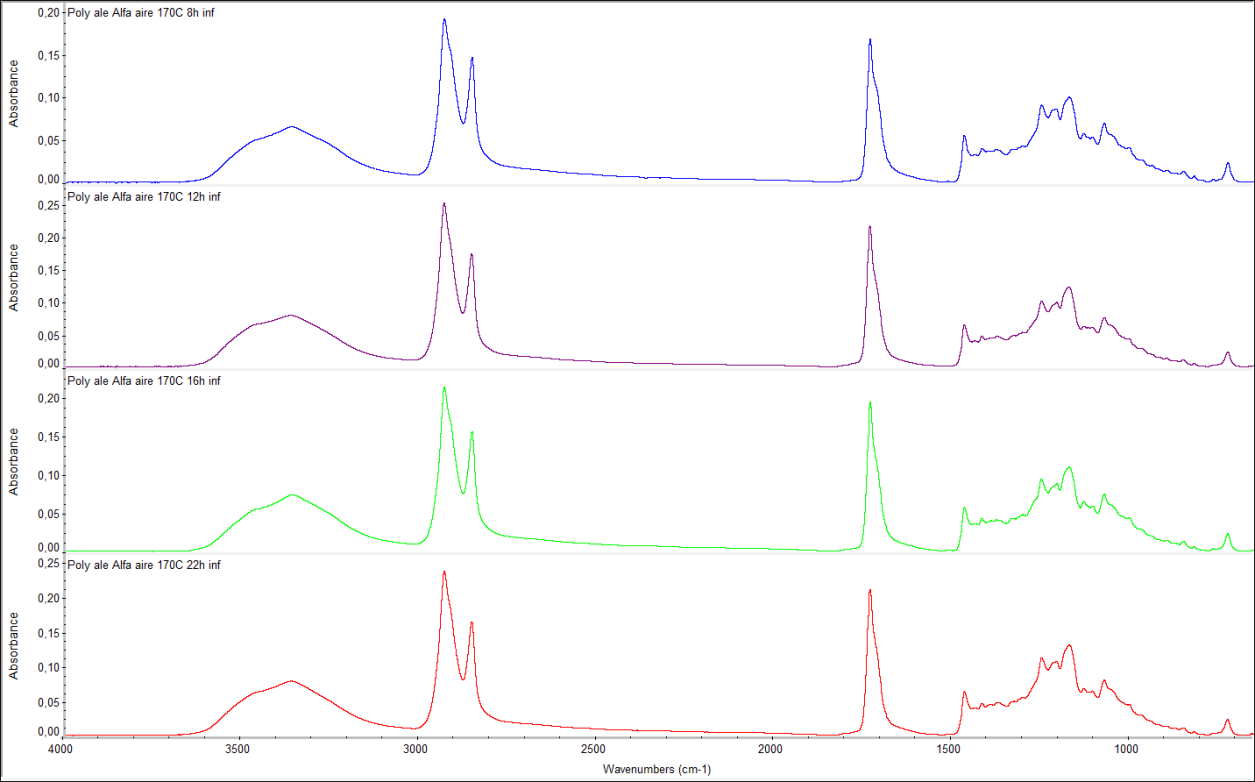


**Figure S3.** ATR-FTIR spectra of the air preserved side of the series shown in figure S1. As above, the formation of the ester is confirmed and indicated a bulk reaction. The presence of progression bands in the 800-1400 cm^-1^ region and the structure of the ν(O-H) (3100-3600 cm^-1^) reveals a higher crystallinity in the absence of oxygen. Accordingly, no oxidized species and no modification of the ν(C=O)/ν(CH_2_) band area ratio are observed.


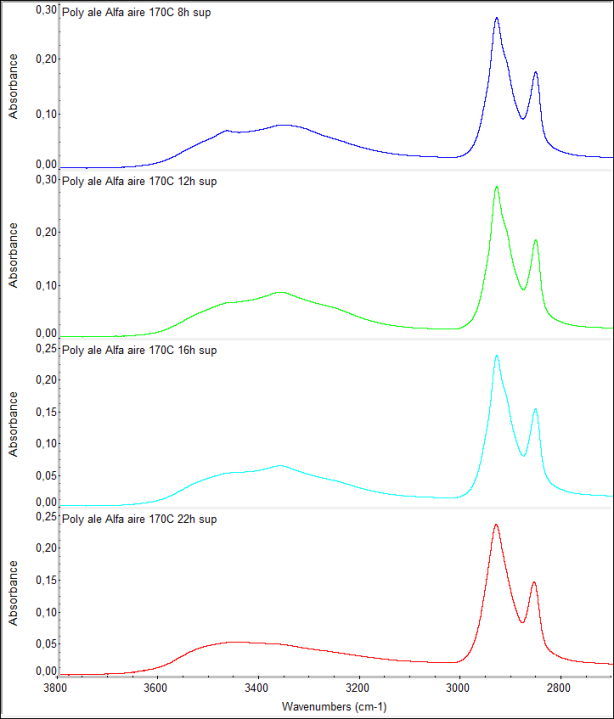

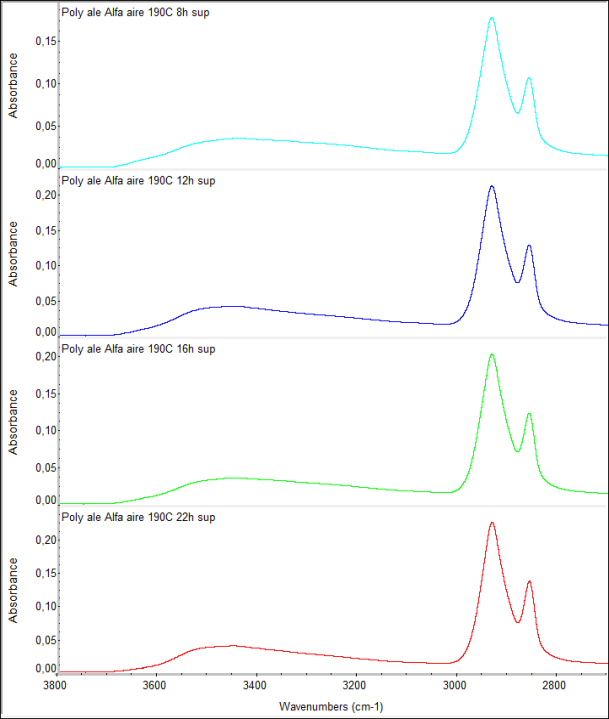


**Figure S4.** 3800-2700 cm^-1^ ATR-FTIR region of the air exposed side of polyaleuritate films prepared at 170°C and 190°C without catalyst. The loss of structure of the OH stretching band is representative for the amorphization process while the intensity loss is consistent with the hydroxyl oxidation in the diol cleavage mechanism proposed.


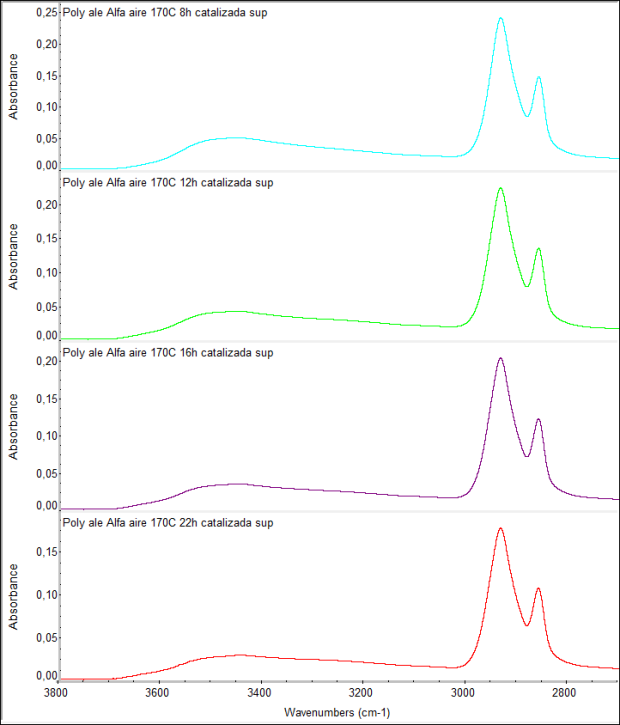

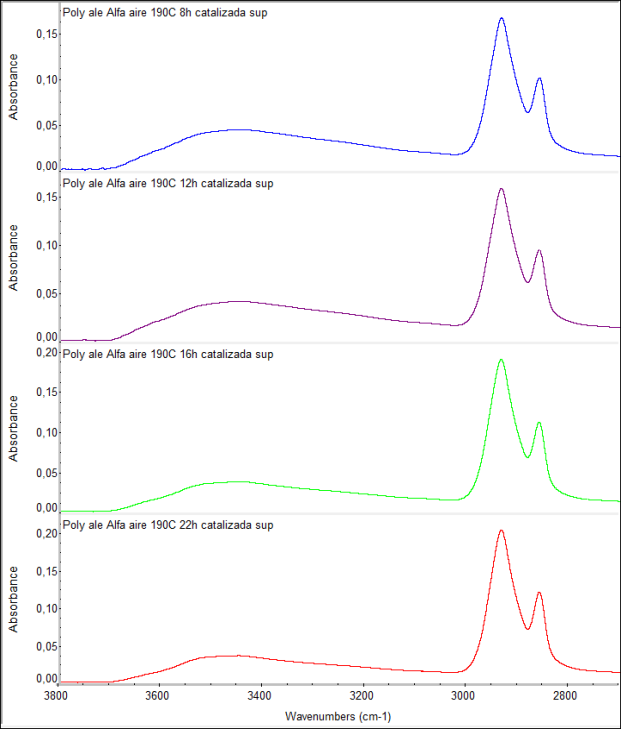


**Figure S5.** Analogous ATR-FTIR data for the catalyzed series. It is clearly observable that both the amorphization and the hydroxyl oxidation are enhanced by the presence of the catalyst. It has to be taken into account that quantitative results cannot be obtained from the area of the hydroxyl stretching band because of the contribution of environmental water to the ATR spectra.


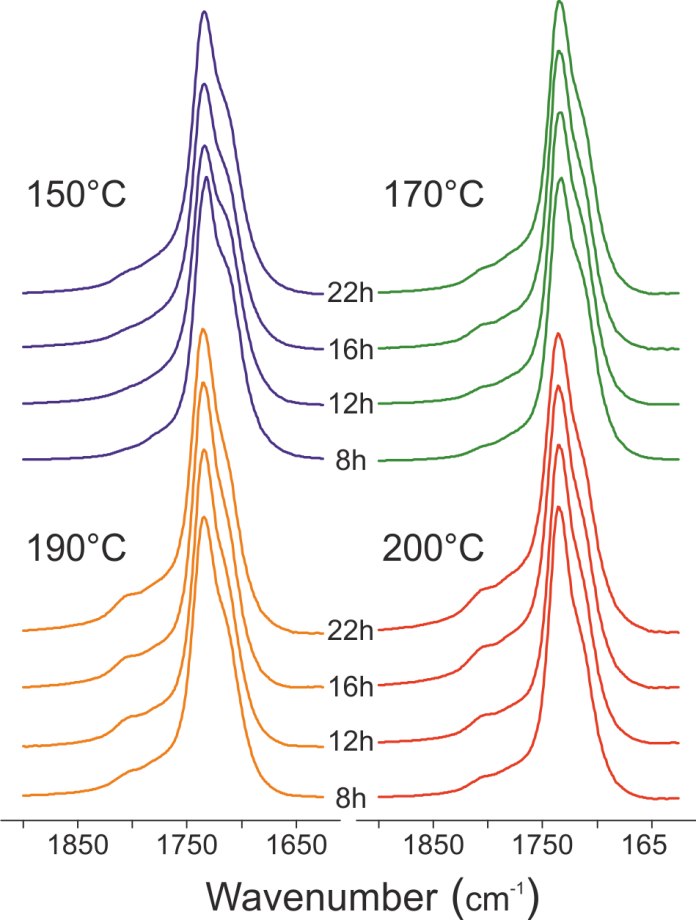


**Figure S6.** Transmission IR spectra of the carbonyl stretching region for the non catalyzed series of polyaleuritates prepared in air. The formation of peroxyesters and diacyl peroxides as oxidation by-products is confirmed by bands at 1803 and 1773 cm^-1^, respectively. Their formation is favored at higher temperature and longer reaction time. IR transmission data provides a realistic evaluation of the overall oxidation extent. On the other side, ATR-IR analysis is more appropriate to characterize the oxidation process at the air exposed side of polyaleuritates.


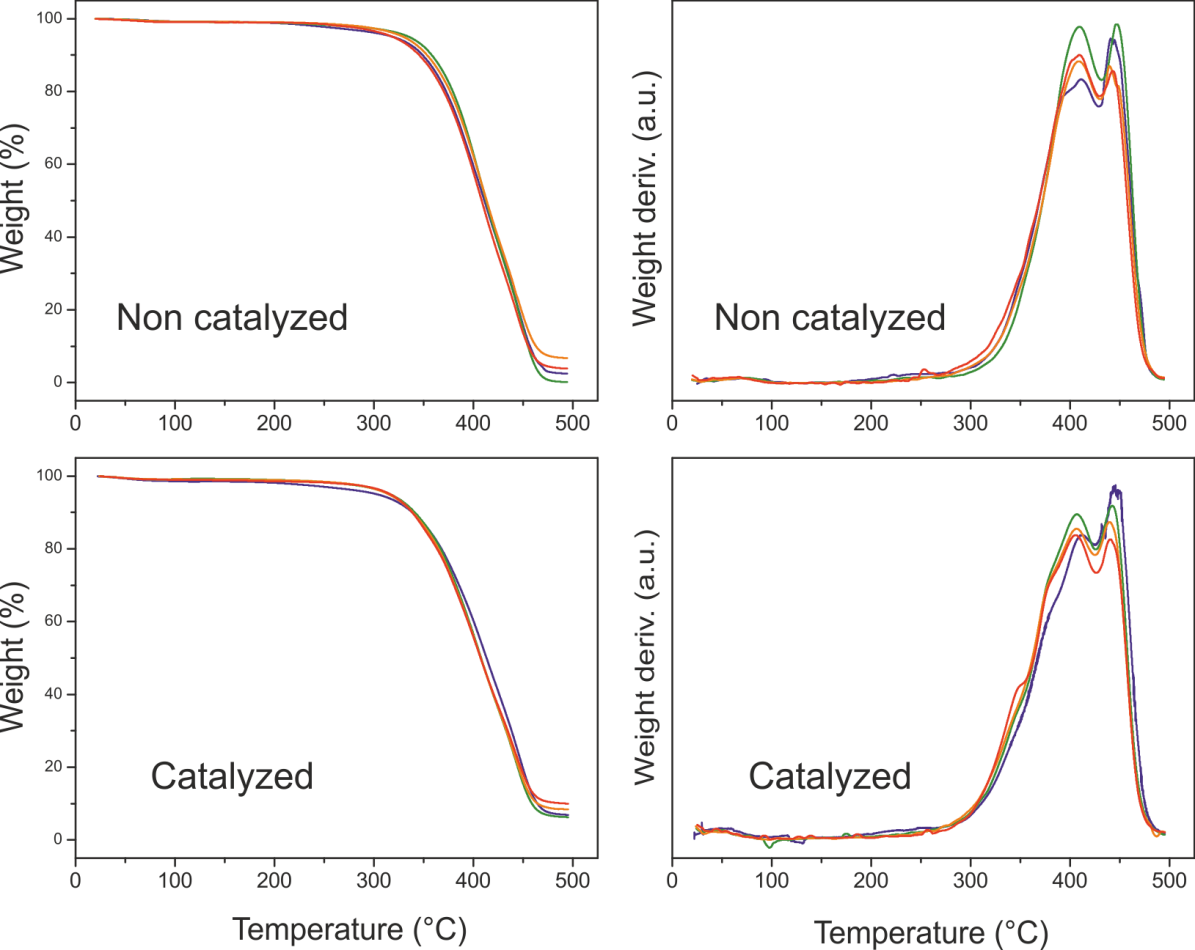


**Figure S7.** TGA thermograms (left) and derivative traces (right) for polyaleuritates prepared with (bottom) and without (top) Ti(OiPr)_4_ catalyst at 150 (blue), 170 (green), 190 (orange) and 200°C (red) for 22h. No meaningful differences can be observed in the thermal stability of polyaleuritates despite the different preparation conditions used.


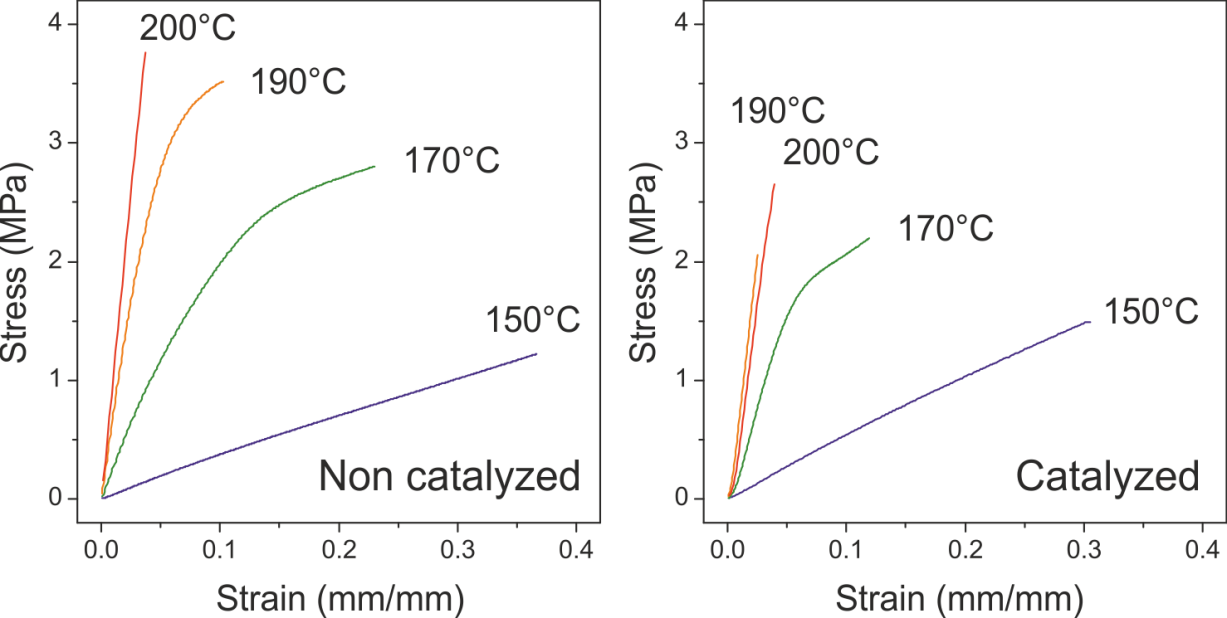


**Figure S8.** Stress-Strain curves for polyaleuritates films synthesized for 22h at the indicated reactions conditions in air. A progressive stiffening is observed as the reaction temperature is raised and this process is faster for the Ti(OiPr)_4_ catalyzed series. Brittling is also favored in the presence of the catalyst.


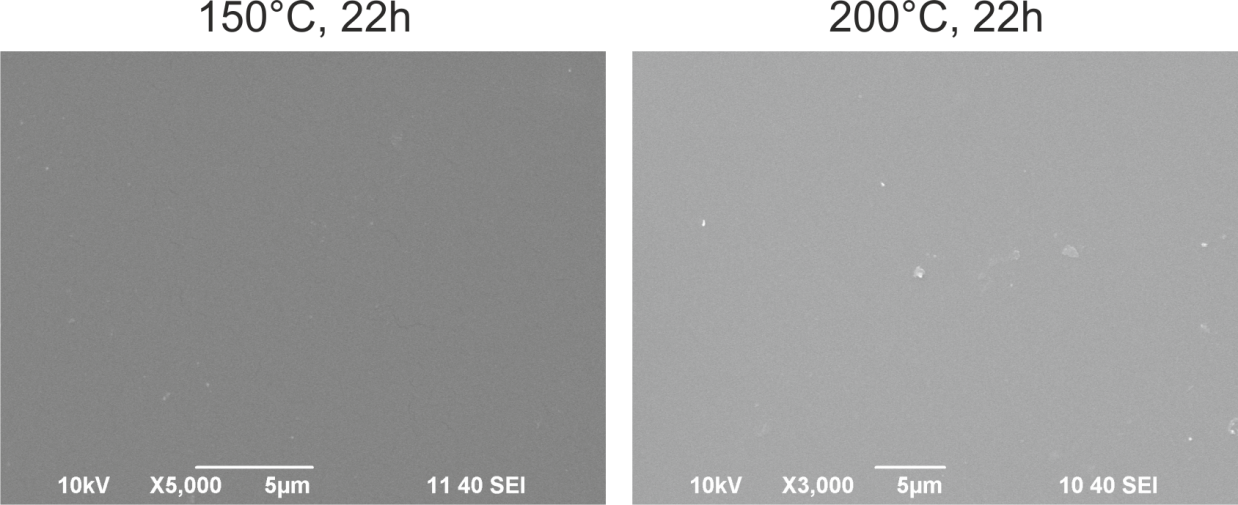


**Figure S9.** SEM images of the air exposed side of polyaleuritates prepared at 150°C and 200°C for 22h. Surface roughness was too low to be resolved by this technique.
